# SHMT2 promotes papillary thyroid cancer metastasis through epigenetic activation of AKT signaling

**DOI:** 10.1038/s41419-024-06476-1

**Published:** 2024-01-25

**Authors:** Min Sun, Mingjian Zhao, Ruowen Li, Yankun Zhang, Xiaojia Shi, Changyuan Ding, Chunhong Ma, Jinghui Lu, Xuetian Yue

**Affiliations:** 1 Department of General Surgery, Qilu Hospital, Cheeloo College of Medicine, Shandong University, Jinan, 250012 China; 2 Key Laboratory for Experimental Teratology of Ministry of Education, Key Laboratory of Infection and Immunity of Shandong Province and Department of Immunology, School of Basic Medical Sciences, Cheeloo Medical College of Medicine, Shandong University, Jinan, 250012 China; 3 Department of Cell Biology, School of Basic Medical Sciences, Cheeloo Medical College of Medicine, Shandong University, Jinan, 250012 China

**Keywords:** Oncogenes, Metastasis

## Abstract

Cancer cells alter their metabolism and epigenetics to support cancer progression. However, very few modulators connecting metabolism and epigenetics have been uncovered. Here, we reveal that serine hydroxymethyltransferase-2 (SHMT2) generates S-adenosylmethionine (SAM) to epigenetically repress phosphatase and tensin homolog (PTEN), leading to papillary thyroid cancer (PTC) metastasis depending on activation of AKT signaling. SHMT2 is elevated in PTC, and is associated with poor prognosis. Overexpressed SHMT2 promotes PTC metastasis both in vitro and in vivo. Proteomic enrichment analysis shows that AKT signaling is activated, and is positively associated with SHMT2 in PTC specimens. Blocking AKT activation eliminates the effects of SHMT2 on promoting PTC metastasis. Furthermore, SHMT2 expression is negatively associated with PTEN, a negative AKT regulator, in PTC specimens. Mechanistically, SHMT2 catalyzes serine metabolism and produces activated one-carbon units that can generate SAM for the methylation of CpG islands in PTEN promoter for PTEN suppression and following AKT activation. Importantly, interference with PTEN expression affects SHMT2 function by promoting AKT signaling activation and PTC metastasis. Collectively, our research demonstrates that SHMT2 connects metabolic reprogramming and epigenetics, contributing to the poor progression of PTC.

## Introduction

Thyroid cancer (THCA) is one of the most common endocrine tumors, and its incidence has been increasing worldwide in decades [1]. Papillary thyroid cancer (PTC) is the most common subtype of thyroid cancer, and has a favorable prognosis and low death rate [2].

However, around 30% to 40% of PTC metastasize to regional lymph nodes and about 1% to 4% of PTC patients may occur distant metastasis [3–5]. The lungs and the bones are the most common sites for distant metastasis [5], which caused dramatic reducing of the survival rate. Especially, a 5-year survival rate decreased from 77.6% in patients with single-organ metastasis to15.3% in patients exhibiting multi-organ metastasis [6]. All these findings indicate that metastasis is the major obstacle for thyroid cancer therapy. Therefore, illustrating the underlying molecular mechanism of PTC metastasis is essential for effectively clinical therapy.

Reprograming of metabolic pathways has been successfully validated to one of the core sets of cancer hallmarks [7]. Serine metabolism plays a critical role in cancer progression, contributing to nucleotide synthesis, methylation reactions and generation of NADPH for redox balance [8]. Amplification of the serine synthesis pathway enzymes has been found in many types of cancer, including hepatocellular carcinoma, colorectal and breast cancer [9–11]. Serine hydroxymethyltransferase 2 (SHMT2) is a key enzyme in serine catabolism existing in mitochondria that converts serine to glycine and produces a one-carbon unit that yields S-adenosylmethionine (SAM) for methylation [12]. This enzyme is highly expressed in more than 85% of cancer types compared with normal tissues [13], induced an increased flux of serine catabolism and one-carbon cycle. Highly SHMT2 expression promotes malignant behavior of cancer cells through multiple biological processes, including cell proliferation, chemoresistance, angiogenesis, migration and invasion [14–16]. Clinical research has shown that SHMT2 is highly expressed in malignant thyroid tissues compare to normal tissues and is associated with poor clinical outcomes [17], but the functions and underlying mechanisms of SHMT2 in PTC remain unclear. Thus, elucidating the functions and molecular mechanism of SHMT2 in PTC would benefit its diagnosis and therapy.

AKT (Protein kinase B) signaling pathway is frequently activated in many human cancer types, such as breast, prostate, lung, and gastric cancer [18, 19]. Many oncogenes activate AKT signaling pathway resulting in cell proliferation, anti-apoptosis, tumorigenesis, chemoresistance, and metastasis [20–22]. Mounting clinical and experimental data have demonstrated that AKT signaling pathway plays a critical role in PTC formation and progression [23, 24]. However, the molecular mechanisms underlying AKT signaling activation in PTC are still not fully understood. The most prominent negative upstream regulator of AKT is PTEN (phosphatase and tensin homolog) which inhibits AKT activity by suppressing its phosphorylation [20, 23]. Previous reports have shown that expression of PTEN was decreased in PTC caused by mutations or promoter methylation [25, 26]. However, the upstream modulator that regulates methylation of PTEN promoter is still unclear. SAM, a product of SHMT2-catalyzed serine metabolism, is a universal biological cofactor that provides a methyl group to various biomolecules, including DNA, RNA, and proteins [27]. Whether SHMT2-induced SAM production contributes to methylation of PTEN promoter and subsequent AKT signaling activation in PTC has not been elucidated.

Here, we found that elevation of SHMT2 augmented the activation of AKT signaling through the repression of PTEN depending on DNA methylation in PTC. Our study verified that SHMT2 modulates metabolic reprogramming and epigenetics in PTC. These findings suggest potential therapeutic strategies for PTC.

## Results

### SHMT2 is elevated in PTC and associated with poor prognosis

**Fig. 1:**
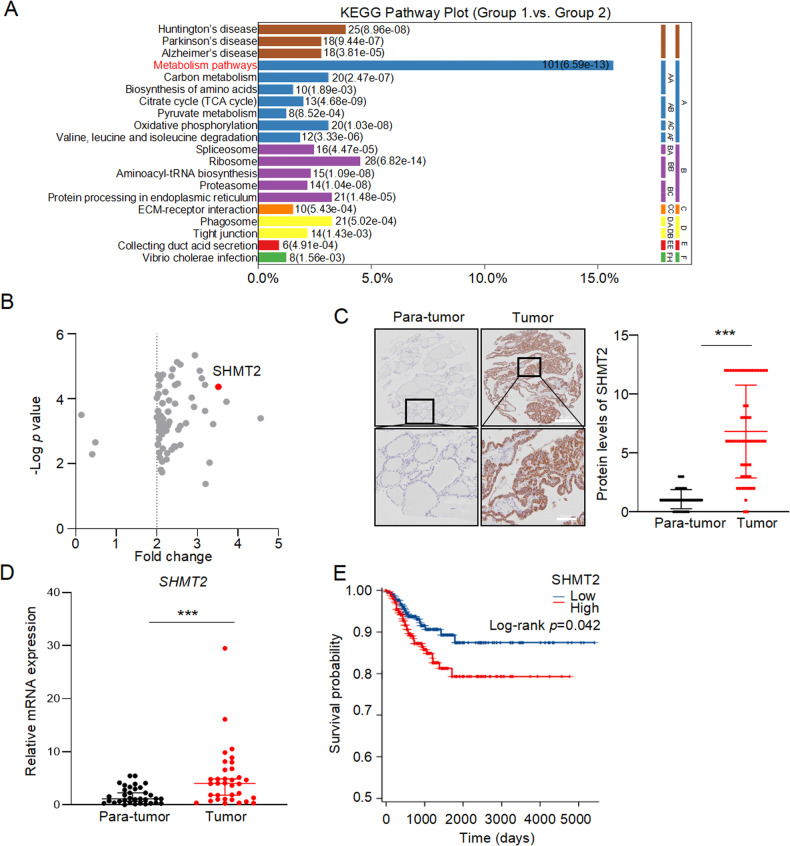
Elevation of SHMT2 was associated with poor prognosis in PTC. **A** KEGG enrichment analysis was performed with the differential proteins from tumor tissues and para-tumor tissues of PTC patients. *n* = 27. **B** Volcano plots showed differently expressed mitochondrial located metabolic genes in PTC. Short dash line: Fold change ≥ 2. Redpoint: SHMT2. **C** The expression of SHMT2 were detected in tumor and para-tumor tissues by IHC staining. Left: representative histological micrographs. Scale bar: 200 μm (up panel), 100 μm (bottom panel). Right: statistics of PTC microarray. Data presented as mean ± SD, *n* = 58. ***: *p* < 0.001. **D** Relative mRNA levels of SHMT2 were examined by RT-qPCR in tumor and para-tumor tissues from cohort of Qilu Hospital. Data presented as mean ± SD, *n* = 34. ***: *p* < 0.001. **E** Kaplan-Meier curves showed the survival of individuals with PTC from TCGA database, separating based on the expression of SHMT2.

To identify the potential drivers of papillary thyroid carcinoma (PTC), we further analyzed our previous proteomics data which includes 27 paired clinical samples [28]. Kyoto encyclopedia of genes and genomes (KEGG) pathway analysis revealed that most of differentially expressed proteins were enriched in metabolic pathways (Fig. 1A). Since mitochondria is the central node of metabolism, proteins enriched in metabolic pathways and those located in mitochondria were intersected with the Venn diagram. As shown in Fig. S1A, 51 candidate proteins were identified. Among them, SHMT2 was identified as one of the most significantly changed proteins (Fig. 1B). To confirm these results, we used immunohistochemistry (IHC) to detect SHMT2 expression in PTC tissue microarray. Evidently, SHMT2 protein levels were dramatically elevated in tumor tissues compared to those in para-tumor tissues (Fig. 1C). Consistently, *SHMT2* mRNA levels were higher in tumors than those in para-tumors in Qilu Hospital cohort (Fig. 1D). In addition, TCGA data showed SHMT2 was increased in PTC at transcriptional level (Fig. S1B). Further analysis showed that SHMT2 was highly expressed in PTC tissues from patients aged > 55 (Fig. S1C). Importantly, higher SHMT2 expression was associated with poor prognosis of PTC patients (Fig. 1E). It is worth to note that SHMT2 expression was also elevated in benign thyroid adenomas and anaplastic carcinomas compared to normal tissues (GSE27155) [29] (Fig. S1D). Above all, SHMT2 expression is elevated in PTC and associated with poor prognosis of PTC patients.

### SHMT2 promotes PTC metastasis both in vitro and in vivo

**Fig. 2:**
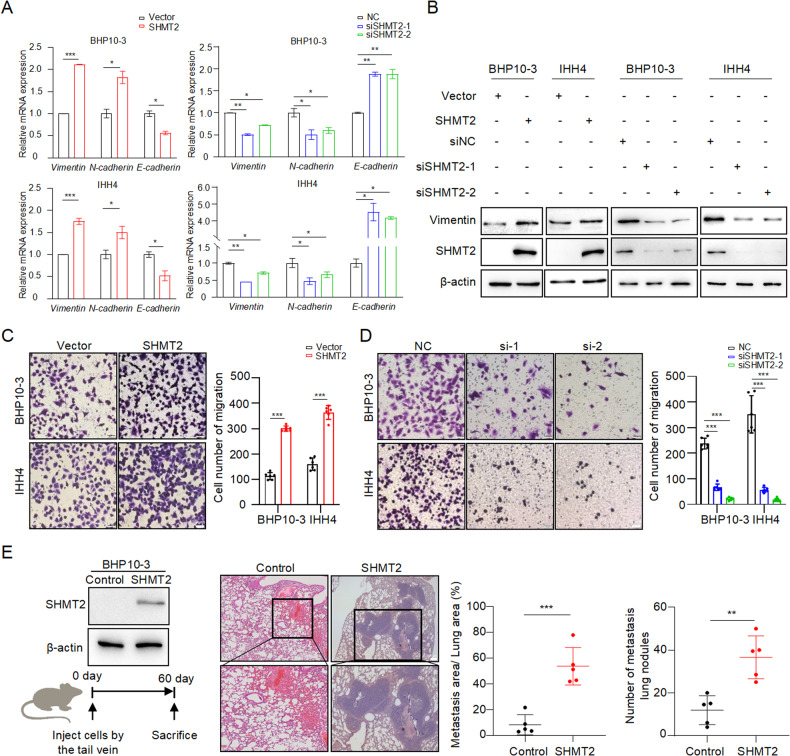
SHMT2 promotes PTC metastasis in vitro and in vivo. **A.** The mRNA levels of EMT markers were determined in BHP10-3 and IHH4 cells with SHMT2 overexpression or knockdown, respectively. Data presented as mean ± SD, *n* = 3. *: *p* < 0.05, **: *p* < 0.01, ***: *p* < 0.001. **B** Protein levels of Vimentin were examined in BHP10-3 and IHH4 cells with SHMT2 overexpression or knockdown, respectively. **C**, **D** Migration assays were performed in PTC cell lines with SHMT2 overexpression (**C**) or knockdown (**D**). Left panel: representative images, scal bar: 50 μm. Right panel: statistical analysis of migrated cells. Data presented as mean ± SD, *n* = 6. ***: *p* < 0.001. **E** Lung metastasis was performed using BHP10-3 cells with SHMT2 overexpression. Left panel: western blot of SHMT2 overexpression (up) and workflow for lung metastasis (bottom). Middle panel: representative H&E images of lung collecting from each group. Scale bar: 100 μm. Right panel: statistical analysis of metastatic area and number of metastatic nodules. Data presented as mean ± SD, *n* = 5, Scale bar: 200 μm. **: *p* < 0.01, ***: *p* < 0.001.

The migration ability was examined in PTC cell lines with SHMT2 manipulation, since metastasis is the major obstacle for PTC therapy. As shown in Fig. S2, the overexpression or knockdown efficiency of SHMT2 was evaluated in BHP10-3 and IHH4 cells. The expression of Vimentin, N-cadherin and E-cadherin, well-known cell migratory markers [30], was detected in PTC cell lines with SHMT2 overexpression or knockdown. The results showed that overexpressed SHMT2 increased *Vimentin* and *N-cadherin* and decreased *E-cadherin* at mRNA levels in BHP10-3 and IHH4 cells. Reciprocally, knockdown of SHMT2 decreased *Vimentin* and *N-cadherin* and increased *E-cadherin* (Fig. 2A). Similarly, SHMT2 levels were positively associated with Vimentin protein levels in PTC cell lines (Fig. 2B). Importantly, transwell assays observed that the number of migrated cells were clearly increased in SHMT2-transfected PTC cells compared to those in control cells (Fig. 2C). Conversely, SHMT2 knockdown dramatically inhibited the migratory ability of PTC cells (Fig. 2D). To further verify these results, BHP10-3 cells with stable overexpressed SHMT2 were established for mouse lung metastasis assays (Fig. 2E, left panel). H&E staining of lung tissues showed that larger metastatic tumor nodes were formed in the mouse lung with injection of SHMT2-overexpressed BHP10-3 than those of control (Fig. 2E, middle panel). Consistently, metastatic area of the mouse lung was significantly larger and number of metastasis tumors was higher in the SHMT2 overexpressed group than those in the control group (Fig. 2E, right panel). Therefore, SHMT2 acts as a metastasis-promoting factor in PTC.

### SHMT2 activates AKT signaling pathway in PTC

**Fig. 3:**
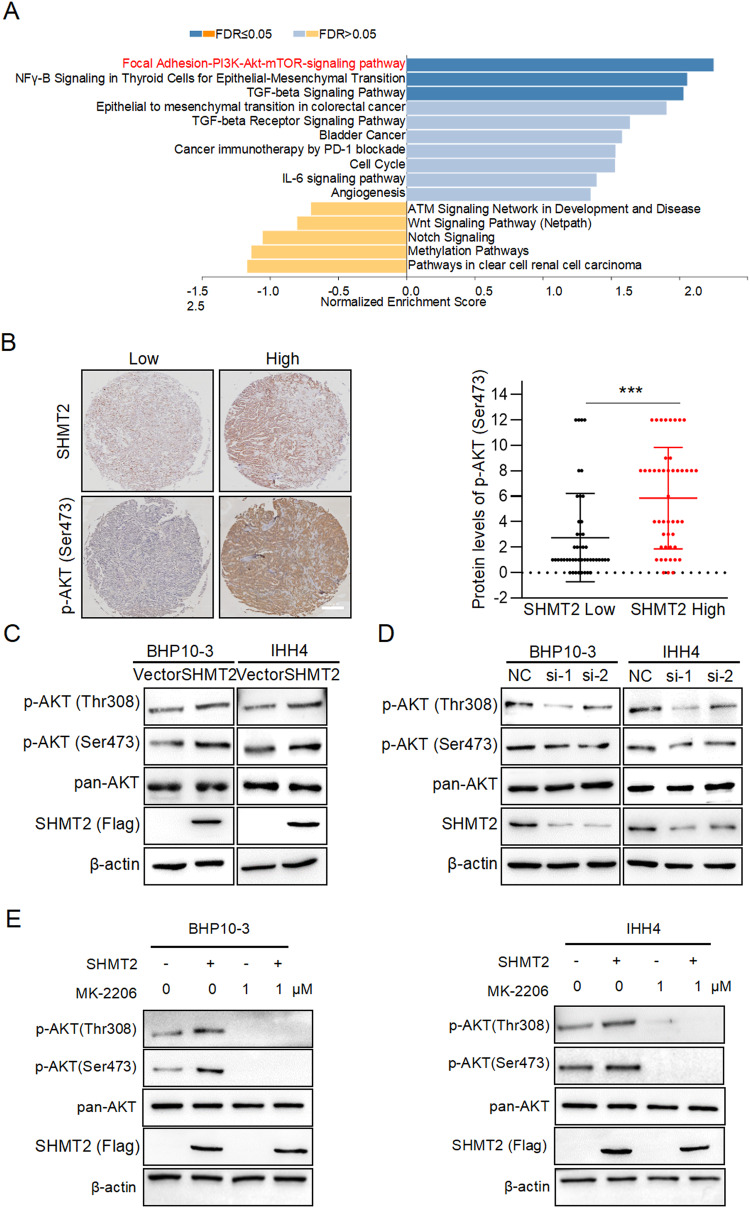
SHMT2 activates AKT signaling pathway. **A** GSEA analysis was performed with the differentially expressed proteins from tumor and para-tumor tissues. **B** Protein levels of p-AKT (Ser473) and SHMT2 were detected by IHC staining in tumor tissues. Left panel: representative histological micrographs of IHC. Scale bar, 200 μm. Right panel: statistical analysis of the correlation of p-AKT (Ser 473) and SHMT2 in tumor tissues. n = 58. ***: *p* < 0.001. **C**, **D** Levels of p-AKT (Ser473 and Thr308) were detected in BHP10-3 or IHH4 cells with SHMT2 overexpression or knockdown by using western blot, respectively. **E** BHP10-3 and IHH4 cells were transfected with SHMT2 and/or treated with MK-2206, individually or simultaneously. Then phosphorylation of AKT was determined in these cells.

To gain insight into the mechanism of SHMT2 promoting PTC metastasis, we further analyzed our previous proteomic data [28]. The results showed that several oncogenic signaling pathways were influenced in tumor tissues. Among them, PI3K-AKT signaling pathway was the most significantly activated in tumor samples (Fig. 3A). This result was confirmed by GSEA (NES = 2.25; *p* = 0.000; FDR = 0.0165) (Fig. S3A). Then, the marker of AKT activation phosphorylated-AKT (Ser473) was examined in PTC specimens. As shown in Fig. S3B, the phosphorylation of AKT (Ser 473) were obviously increased in tumors compared to those in para-tumors. Importantly, the levels of phosphorylated-AKT (Ser 473) were higher in tumor samples with high SHMT2 expression than in those with low SHMT2 expression (Fig. 3B). To verify these findings, SHMT2 was overexpressed or silenced in BHP10-3 and IHH4 cells to detect phosphorylated-AKT, respectively. The results showed that SHMT2 overexpressing increased the protein levels of phosphorylated AKT (Ser 473 and Thr 308), whereas SHMT2 silencing decreased AKT phosphorylation (Fig. 3C, D). Furthermore, treatment with MK-2206, the inhibitor of AKT phosphorylation, decreased the levels of phosphorylated AKT (Ser 473 and Thr 308). Notably, MK-2206 inhibited the SHMT2-induced AKT phosphorylation (Fig. 3E). In summary, these results indicate that SHMT2 enhances AKT phosphorylation and activation in PTC cells.

### Inhibition of AKT pathway blocks SHMT2-induced metastasis of thyroid cancer

**Fig. 4:**
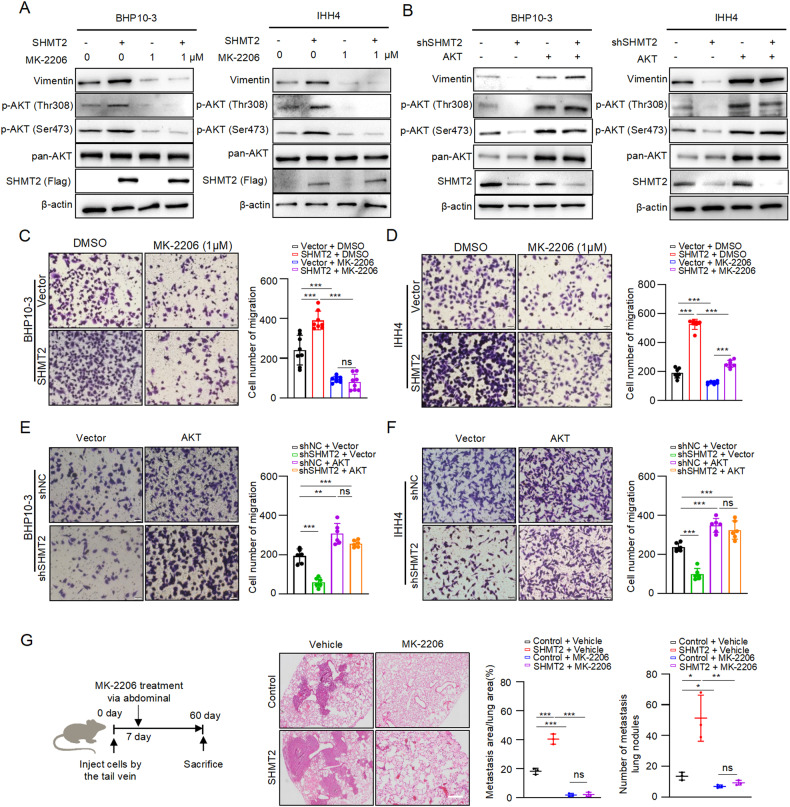
Blocking AKT activation eliminates SHMT2-induced metastasis in thyroid cancer. **A** Protein levels of Vimentin were detected in BHP10-3 or IHH4 cells with SHMT2 overexpression and/or MK-2206 treatment by western blot, respectively. **B** Protein levels of Vimentin were examined in BHP10-3 or IHH4 cells with shSHMT2 and/or AKT transfection by western blot, respectively. **C**, **D** Migration assays were performed in BHP10-3 or IHH4 cells with SHMT2 overexpression and/or MK-2206 treatment, receptively. Left panel: representative micrographs of migrated cells. Scale bar: 50 μm. Right panel: statistical analysis of migrated cells. Data presented as mean ± SD, *n* = 8, *n* = 6. ***: *p* < 0.001, ns: no significant. **E**, **F** Migration assays were performed in BHP10-3 or IHH4 cells with shSHMT2 and/or AKT transfection. Left panel: representative micrographs of migrated cells. Scale bar: 50 μm. Right panel: statistical analysis of migrated cells. Data presented as mean ± SD, *n* = 6. **: *p* < 0.01, ***: *p* < 0.001, ns: no significant. **G** SHMT2 overexpressed and control BHP10-3 cells were injected in node mice via tail vein, followed by treatment with MK-2206 (5 mg/kg) for lung metastasis assays. Right panel: work follow of lung metastasis assays. Middle panel: representative histological micrographs of mice lung. Scale bar: 100 μm. Right panel: statistical analysis of metastasis tumor. Data presented as mean ± SD, *n* = 3. *: *p* < 0.05, **: *p* < 0.01, ***: *p* < 0.001, ns: no significant.

To explore the role of SHMT2-induced activation of AKT signaling in PTC metastasis, we performed the following experiments. As shown in Fig. 4A, consistent with previous results overexpression of SHMT2 increased Vimentin at protein levels in BHP10-3 and IHH4 cells. Notably, treatment with MK-2206 decreased Vimentin expression and abolished SHMT2-induced Vimentin increasing. Reciprocally, knockdown of SHMT2 decreased Vimentin protein, overexpression of AKT up-regulated Vimentin expression and reversed SHMT2 silencing-induced decreasing of Vimentin in BHP10-3 and IHH4 (Fig. 4B). Furthermore, transwell assays showed that SHMT2 enhanced the migratory ability of BHP10-3 and IHH4 cells, and MK-2206 treatment eliminated the effect of SHMT2 on promoting cell migration (Fig. 4C, D). Accordingly, silencing of SHMT2 decreased the number of migrated BHP10-3 and IHH4 cells, while overexpression of AKT promotes cell migration and reversed shSHMT2-induced decrease in the migratory ability (Fig. 4E, F). To verify these results in vivo, MK-2206 was used to treat the mice injected with SHMT2 stably expressed BHP10-3 and control cells for mouse lung metastasis assays (Fig. 4G, left panel). Consistent with the data in vitro, overexpression of SHMT2 dramatically increased the area and number of the mouse lung metastatic tumor nodes, and MK-2206 administration abolished the effect of SHMT2 on enhancing lung metastasis in mice (Fig. 4G, middle and right panel). Collectively, all these data demonstrate that SHMT2 enhances the ability of PTC metastasis by activating AKT signaling in vitro and in vivo.

### SHMT2 activates AKT signaling pathway through repression of PTEN

**Fig. 5:**
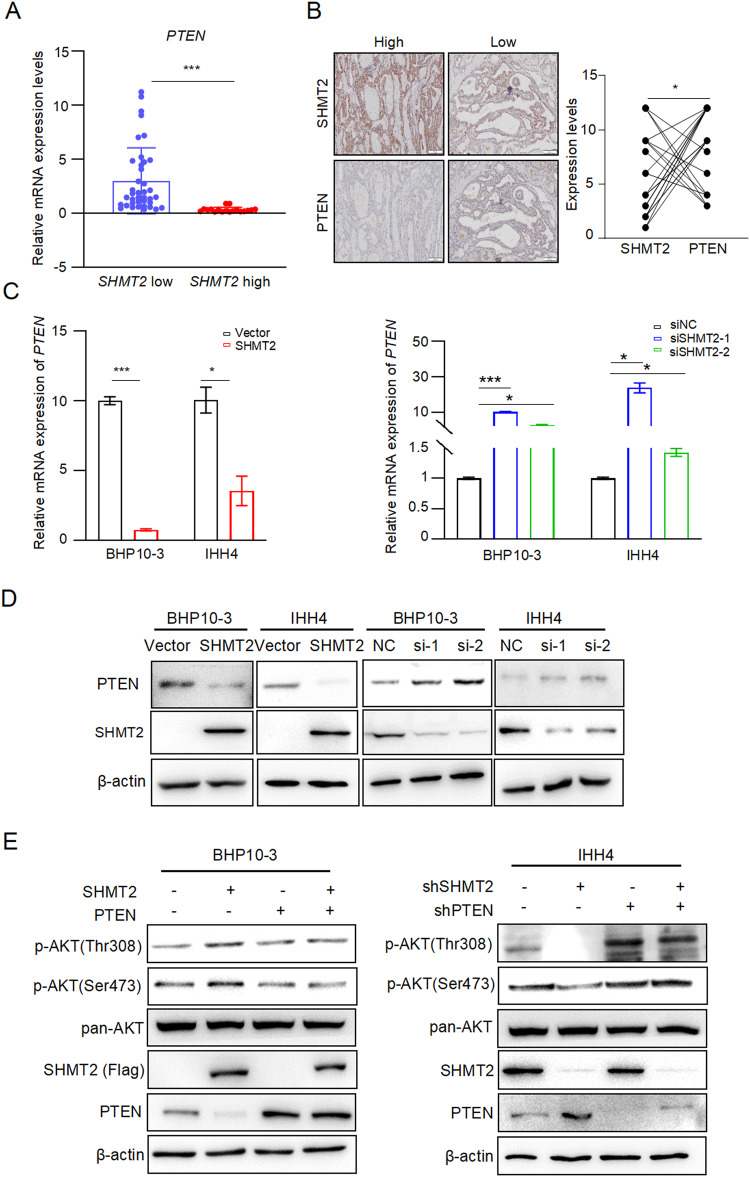
SHMT2 activates AKT signaling through repression PTEN. **A** Relative mRNA levels of *PTEN* were examined in clinical samples grouped by SHMT2 levels. *n* = 58. ***: *p* < 0.001. **B** Protein levels of PTEN and SHMT2 were evaluated in clinical samples to analyze for their correlation. Left panel: representative micrographs of IHC stained with anti-PTEN or anti-SHMT2. Scal bar: 100 μm. Right panel: statistical analysis of PTEN and SHMT2 correlation. *n* = 21. *: *p* < 0.05. **C** Relative mRNA levels of *PTEN* were examined in BHP10-3 or IHH4 cells with SHMT2 overexpression or knockdown. Data presented as mean ± SD. *: *p* < 0.05, ***: *p* < 0.001. **D** Protein levels of PTEN were determined in BHP10-3 or IHH4 cells with SHMT2 overexpression or knockdown. **E** The protein levels of phosphorylated AKT were evaluated in BHP10-3 or IHH4 cells with SHMT2 and/or PTEN transfection by using western blot (left panel). AKT activation were examined in BHP10-3 or IHH4 cells with shSHMT2 and/or shPTEN transfection by detecting p-AKT using western blot (right panel).

In order to elucidate how SHMT2 activates AKT signaling pathway, PTEN, the negative regulator of AKT [31], was examined in human PTC samples. Consistent with previous reports [32, 33], the expression of PTEN was decreased in tumor compared to para-tumor at both protein and mRNA levels (Fig. S4A and S4B). Importantly, *PTEN* mRNA levels were lower in tumor tissues with high SHMT2 expression than in specimens with low SHMT2 levels (Fig. 5A). Consistently, tumor tissues with high SHMT2 expression showed weak intensity of PTEN staining, while strong PTEN staining was observed in tissues with low SHMT2 expression (Fig. 5B). To verify these results, PTEN expression was detected in BHP10-3 and IHH4 cells with SHMT2 overexpression or knockdown, respectively. As shown in Fig. 5C, D, overexpression of SHMT2 decreased PTEN expression at both mRNA and protein levels in BHP10-3 and IHH4 cells. In contrast, the knockdown of SHMT2 increased PTEN expression. Importantly, overexpression of SHMT2 activated AKT, overexpression of PTEN inhibited AKT activation and blocked SHMT2-induced AKT activation (Fig. 5E). Reciprocally, shSHMT2 transfection reduced AKT activation, shPTEN transfection promoted AKT activation and reversed silencing of SHMT2 in inhibiting AKT signaling activation (Fig. 5E). Above all, these results suggest that SHMT2 activates AKT by suppressing PTEN.

### SHMT2 decreases PTEN expression via DNA methylation

**Fig. 6:**
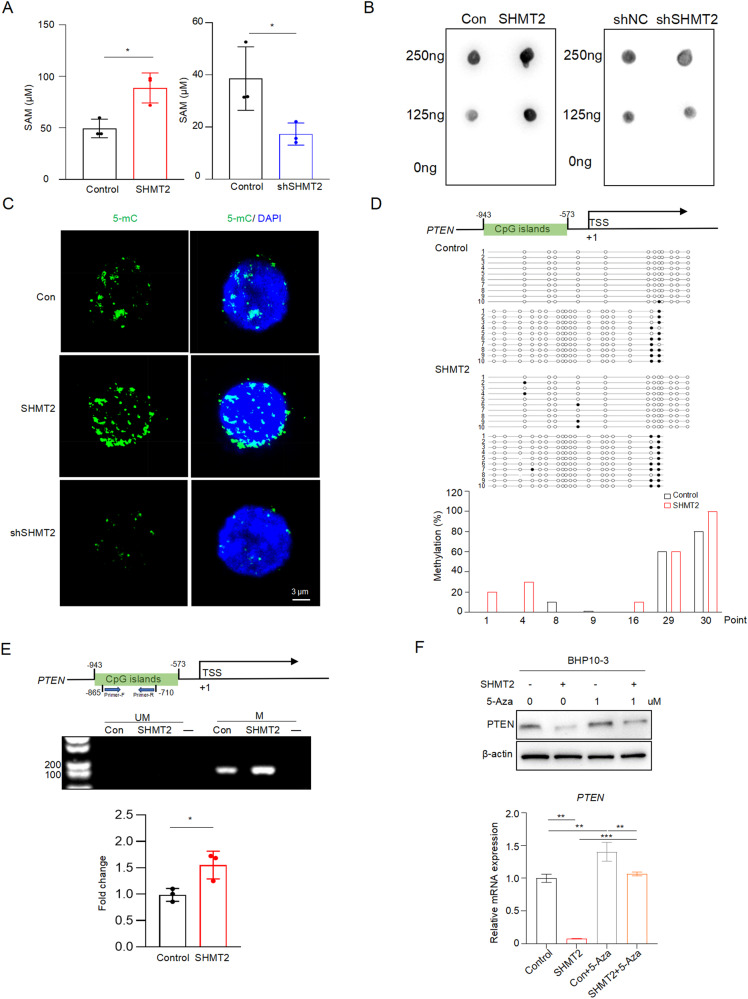
SHMT2 represses PTEN expression by increasing DNA methylation. **A** SAM levels were assessed in BHP10-3 cells with SHMT2 over-expression or knockdown, respectively. Data presented as mean ± SD, *n* = 3. *: *p* < 0.05. **B** Dot blot of DNA was performed using DNA extracted from SHMT2 over-expressed or knockdown BHP10-3 cells. **C** IF staining was performed in BHP10-3 cells with SHMT2 over-expression or knockdown using anti-5mC. Representative images were presented. Green: 5-mC, blue: DAPI. Scale bar: 3 µm. **D** Methylation analysis of PTEN promoter. Up panel: Schematic diagram showed the sequenced CpG islands on PTEN promoter extending from -943 to -573 from TSS. Middle panel: Bisulfite DNA sequencing of PTEN promoter. Each line represents a single clone. Black dot: methylated, white dot: unmethylated. Bottom panel: The graphs showed the methylation percentages of cytosine in the target regions of PTEN promoter. **E** Methylation of PTEN promoter region were determined by MSP. Up panel: Schematic graph showed the location of CpG islands and MSP primers. Middle panel: Representative images of DNA blot. UM: unmethylated, M: methylated. Bottom panel: statistical analysis of methylated DNA. Data presented as mean ± SD, *n* = 3. *: *p* < 0.05. **F** Protein (up panel) and mRNA (bottom panel) levels of PTEN were evaluated in BHP10-3 cells with SHMT2 over-expression and/or 5-Aza treated (1 µM). Data presented as mean ± SD, *n* = 3. **: *p* < 0.01, ***: *p* < 0.001.

Since methylation of PTEN promoter has been reported in thyroid cancer and SHMT2 catalyzes the generation of methyl donor SAM [12, 25, 26], we hypothesized that SHMT2 represses PTEN expression via DNA methylation. Indeed, total SAM levels were increased in SHMT2 overexpressed cells and decreased in SHMT2 knockdown cells (Fig. 6A). Consequently, DNA dot blot displayed that the intensity of 5-mC was enhanced in SHMT2 overexpressed cells and reduced in SHMT2 knockdown cells (Fig. 6B), indicating that SHMT2 levels affect global DNA methylation. Similarly, IF staining showed that the nuclear intensity of 5-mC was positively associated with SHMT2 levels in BPH10-3 cells (Fig. 6C). To clearly display methylation status of PTEN promoter, we performed methylation sequencing in BHP10-3 cells with SHMT2 overexpression. As shown in Fig. 6D, SHMT2 overexpressed cells displayed higher probability of PTEN promoter methylation, presented as increased methylation of the CpG islands extending from -943 to -573 from the transcriptional start site (TSS). To verify the sequencing data, we performed methylation-specific PCR (MSP) using specific primers targeting on these CpG islands (from -865 to -710 from TSS) in BHP10-3 cells with SHMT2 overexpression. The results showed that SHMT2 overexpression increased the methyl modification on CpG islands located in PTEN promoter (Fig. 6E). Furthermore, treatment with 5-azacytidine (5-Aza), the inhibitor of DNA methylation, eliminated the inhibition of SHMT2 on PTEN expression at both mRNA and protein levels (Fig. 6F). Taken together, above results demonstrate that SHMT2 inhibits PTEN expression by enhancing methylation of CpG islands in its promoter.

### SHMT2 accelerates PTC metastasis by inhibiting PTEN expression

**Fig. 7:**
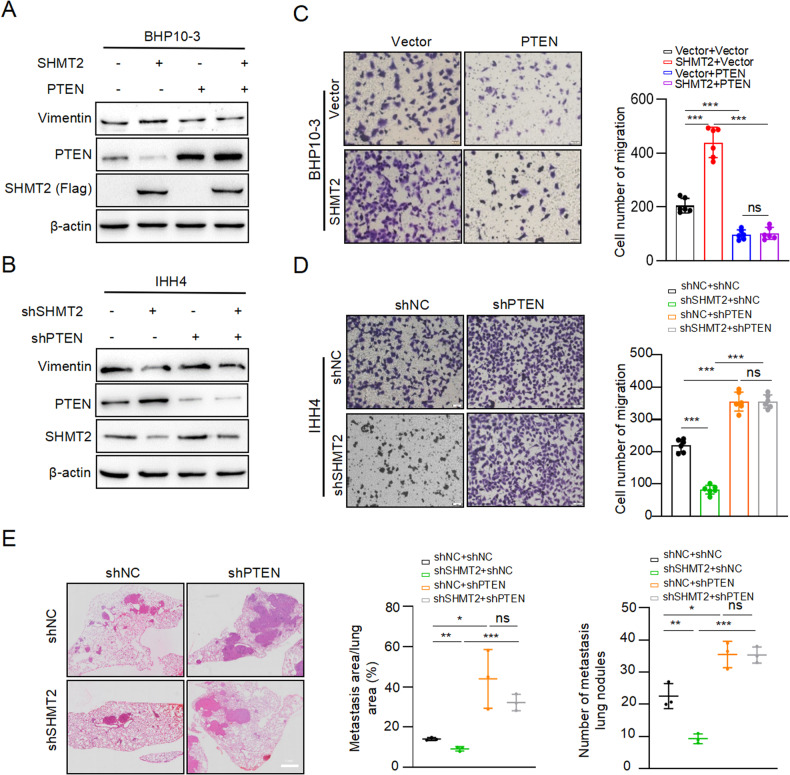
SHMT2 promotes PTC metastasis through repression of PTEN. **A**, **B** Protein levels of Vimentin were determined in BHP10-3 cells with SHMT2 and/or PTEN manipulation by using western blot, respectively. **C**, **D** Migration assays were performed in BHP10-3 cells with SHMT2 or PTEN manipulation, respectively. Left panel: representative micrographs of migrated cells. Scal bar: 50 µm. Right panel: statistical analysis of migrated cells. Data presented as mean ± SD, *n* = 6. ***: *p* < 0.001, ns: no significant. **E** Lung metastasis assays were performed with BHP10-3 cells with indicated manipulation of SHMT2 and PTEN. Left panel: representative images of H&E staining for mice lung. Scal bar: 1 mm. Right panel: statistical analysis of metastasis tumor. Data presented as mean ± SD, *n* = 3. *: *p* < 0.05, **: *p* < 0.01, ***: *p* < 0.001, ns: no significant.

We examined the role of PTEN in SHMT2-drived PTC metastasis. Vimentin protein levels were examined in PTC cell lines with SHMT2 and/or PTEN manipulation, respectively. The results showed that SHMT2 overexpression increased Vimentin protein level, and PTEN transfection abolished SHMT2-induced increasing in Vimentin expression (Fig. 7A). Reciprocally, knockdown of SHMT2 decreased Vimentin expression, and PTEN silencing rescued SHMT2 knockdown-induced decreasing of Vimentin protein levels (Fig. 7B). Consequently, transwell assays displayed that SHMT2 promoted migration of BHP10-3 cells, and PTEN transfection blocked SHMT2-induced increasing of BHP10-3 cells migration (Fig. 7C). Knockdown of SHMT2 in IHH4 cells reduced the number of migrated cells, and PTEN silencing reversed shSHMT2-induced decreasing of migrated cells (Fig. 7D). Furthermore, four groups of stable BHP10-3 cells, including shNC/shNC, shSHMT2/shNC, shNC/shPTEN, and shSHMT2/shPTEN, were injected into mice via tail vein for mouse lung metastasis assays. Consistent with the in vitro data, shSHMT2 decreased the area of mouse lung metastatic tumor nodes, shPTEN increased the area of mouse lung metastatic tumor nodes. Notably, shPTEN reversed the effect of shSHMT2 on the inhibition of mouse lung metastatic tumor nodes formation (Fig. 7E). Overall, SHMT2 accelerates PTC metastasis through inhibition of PTEN, indicating that SHMT2-PTEN-AKT axis plays an important role in promoting PTC metastasis.

## Discussion

Papillary thyroid cancer (PTC) is highly susceptible to cervical lymph node and even distant metastasis, leading to a poor prognosis of patients [34]. Therefore, it is important to elucidate the underlying molecular mechanisms of PTC metastasis. Here, we found that the key metabolic enzyme SHMT2 was elevated in patients with PTC, and was positively associated with poor prognosis. Functional assays showed that SHMT2 altered the expression of EMT makers, such as Vimentin, N-cadherin and E-cadherin, and promoted migration of PTC cells both in vitro and in vivo. Mechanistically, SHMT2 activates AKT signaling pathways by inhibiting PTEN expression depending on DNA methylation. Furthermore, interfering PTEN expression or AKT activation affected SHMT2 ability of promoting PTC metastasis. Thus, our study not only validated the elevation of SHMT2 in PTC, but also revealed its function and underlying mechanism in promoting PTC metastasis. These would be beneficial for PTC diagnosis and therapy.

Metabolic reprogramming is a hallmark of cancer, in which serine metabolism is frequently altered [7]. Active serine metabolism provides ample raw material for tumor biological processes, such as NADPH generation for ROS scavenging, substrates for protein synthesis, and one carbon for nucleotide synthesis and DNA/RNA/protein methylation modification [15]. SHMT2 is a key enzyme in serine metabolism, and is highly expressed in a variety of tumors, including breast cancer, lung cancer, B-cell lymphoma and gastric cancer [35–38]. Here, our results showed that SHMT2 is elevated in PTC and is positively associated with poor prognosis of patients. Importantly, SHMT2 overexpression promoted migration of PTC cells, which might reduce the efficiency of PTC treatment. Similar to our findings, many reports have demonstrated that SHMT2 promotes metastasis in human colorectal, breast, and lung tumors [39–41]. Recently, the cytoplasmic isoform of SHMT2 was identified as a driver of tumor progression [42, 43]. These findings illustrate that SHMT2 plays an oncogenic role in many types of tumors. Consistently, our data demonstrated that SHMT2 promotes PTC migration and is associated with poor prognosis in PTC patients.

The PI3K-AKT signaling pathway is one of the highly activated classical signaling pathways in tumors [44]. AKT is a core factor in this signaling pathway, and its dysregulation plays a crucial role in a variety of tumors, such as liver, lung, colon, and breast tumors [45–48]. Numerous studies have reported that AKT promotes tumor progression by inhibiting apoptosis, promoting tumor proliferation, metastasis and reprogramming tumor metabolism [44]. In this study, PI3K-AKT signaling was activated in PTC. Consequently, clinical data showed that high SHMT2 expression was associated with increased AKT phosphorylation (Ser473), indicating that SHMT2 promotes AKT signaling activation. AKT is regulated by a variety of factors. Phosphorylation of two regulatory residues in the AKT PH domain (Thr308 in the activation segment and Ser473 in the hydrophobic matrix) can be activated by AKT upon stimulation factors such as FGF, VEGF, PDGF and EGF [49]. Our results showed that ectopic expression of SHMT2 increased the protein levels of phosphorylated AKT (Ser 473 and Thr 308), whereas knockdown of SHMT2 decreased AKT phosphorylation. Furthermore, MK-2206, a well-known inhibitor of ATK activation, blocked SHMT2-induced AKT activation and PTC cells migration. Above all, our findings identified SHMT2 as a new activator of AKT signaling pathways in PTC.

Crosstalk between metabolic reprogramming and epigenetics has been evident in tumors, such as IDH1 (isocitrate dehydrogenase1)-related histone and DNA methylation, and Sirtuin family proteins-related histone acetylation [50–52]. SHMT2 catalyzes serine catabolism to generate methyl donors involved in DNA, RNA and protein methylation [12, 53]. For example, SHMT2 induces changes in DNA and histone methylation patterns leading to promoter silencing of tumor suppressor genes in lymphoma [54]. In this study, our data demonstrated that SAM levels were increased in SHMT2 overexpressed cells, which was associated with high 5-mC levels and increased methylation levels of PTEN promoter, leading to decreased PTEN expression and activation of AKT signaling. Previous research had reported the loss or reduction of, as well as inappropriate subcellular compartmentalization of PTEN in PTC [55–57]. PTEN is a classical negative regulator of AKT, which inhibits the activation of AKT signaling pathway [58]. PTEN inhibits AKT phosphorylation by converting PIP3 to PIP2 via its lipid phosphatase activity [59]. Our data demonstrated that SHMT2 reduced PTEN levels by increasing methylation of its promoter to activate AKT signaling, leading to migration of PTC cells. Therefore, our study illustrates a new underlying molecular mechanism of PTEN reduction and AKT activation in PTC, and provides new evidence for the crosstalk between metabolic reprogramming and epigenetic modifications in tumor progression.

In summary, our study demonstrated that the elevation of SHMT2, a key enzyme in serine metabolism, significantly promoted PTC metastasis via activation of AKT signaling pathway. SHMT2 activates AKT signaling pathway through repression of PTEN depending on DNA methylation. This study elucidates a new underlying molecular mechanism of PTC metastasis and provides new evidence for crosstalk between tumor metabolic reprogramming and epigenetics. In this study, we clarified function of SHMT2 in PTC cell lines and specimens. It will be more helpful to verify these findings in other subtypes of thyroid cancer in the future studies.

## Materials and methods

### Tissue microarray and human samples

Clinical samples were obtained from Qilu Hospital of Shandong University. Till October 2019, 48 consecutive patients who underwent total thyroidectomy or lobectomy lymph node dissection for the detection of thyroid nodules or suspected PTC were included, including 39 females and 9 males, aged (45.1 ± 8.5) years. The preoperative diagnosis or suspicion of malignancy was made in 43 patients, all of whom underwent fine needle aspiration (FNA) and were staged according to Bethesda. FNA was unsuccessful or rejected in the remaining 5 patients. Nine participants with diabetes, hyperlipidemia, hypertension, allergic or other metabolic disorders, or a history of thyroid surgery or hormone therapy were excluded from the study. Seven patients had specimens too small for subsequent histopathological examination. Tissue samples were obtained during surgery and immediately frozen in liquid nitrogen and stored at −80 °C until proteomic testing was performed. Papillary thyroid tumor microarrays were purchased from Shanghai Core Ultra Biotechnology (HThyP120CS02). Detail patient information has been presented in our previous work [28].

This study was reviewed by the Ethics Committee of Qilu Hospital of Shandong University and followed ethical standards. And informed consent was obtained from all subjects.

### Cell lines and cell culture

Human thyroid cancer cell line BHP10-3 was gained from Qilu Hospital and IHH4 was purchased from American Type Culture Collection. All cells were maintained at 37 °C, and 5% CO_2_ in RPMI-1640 (C11875500BT, GIBCO) supplemented with 10% fetal bovine serum (FBS) (10270106, GIBCO) and penicillin-streptomycin-glutamine (100×) (10378016, GIBCO). BHP10-3 cell lines with stable knockdown of SHMT2 (LV-shSHMT2) and stable overexpression of SHMT2 (LV-SHMT2) and control cell lines were established and preserved in our laboratory. Briefly, lentiviruses infected cell pools were selected using 1 μg/mL puromycin (S7417, Selleck) for 2 weeks. The overexpressed and knockdown efficiency was probed by RT-qPCR or western blot analysis.

### Primers, siRNAs and plasmids

The primers and siRNA oligos targeting SHMT2 and PTEN were obtained from Sangon Biotech (Shanghai, China). The sequences of the primers and siRNA oligos were shown in Supplementary Table. Flag-tagged SHMT2 plasmid were constructed and purchased from Miaolingbio (Wuhan, China). LV-SHMT2 and LV-shSHMT2 purchased from Genechem (Shanghai, China), using for generation of viruses through a lentivirus-mediated delivery system.

### Transfections

Cells were transfected with various plasmids and siRNA oligos with Lipofectamine 2000 (11668-027, Invitrogen) according to the manufacturer’s protocol. Assays were conducted 48 h post-transfection.

### RNA extraction and real-time quantitative PCR (RT-qPCR)

Total RNAs were prepared using a Gene JET RNA Purification Kit (k0731, Thermo Scientific). cDNAs were prepared with a HiScript II Reverse Transcriptase (Glycerol-free) (RL201-01, Vazyme Biotech) and processed for RT-qPCR (Cham Q Universal SYBR qPCR, Vazyme Biotech) using the primers listed in Supplementary Table.

### Migration assay

For cell migration, 4×10^5^ BHP10-3 cells / 8×10^5^ IHH4 cells in 200 μL of serum-free medium were seeded in an 24-well plate chamber insert (354578, Corning Life Sciences), with medium containing 10% FBS at the bottom. The cells were incubated for 24 h and then fixed with methanol for 10 min. After washing 3 times with PBS, the cells were stained with 0.5% crystal violet blue for 5 min and then washed with double-distilled H_2_O. Cells on the upper surface were removed with a wet swab. The stained migrated cells were examined under the microscope and were calculated via Image J.

### Western blot and antibodies

Cells and tissues were lysed in NP-40 lysis buffer with 1% PMSF for 30 min to obtain proteins. After electrophoresis in PAGE, target proteins were marked by antibodies as bellow: SHMT2 (33443 S, Cell Signaling Technology), PTEN (9188 S, Cell Signaling Technology), Vimentin (10366-1-AP, Proteintech), β-actin (66009-1-lp, Proteintech), Phospho-Akt (Ser473) (4060, Cell Signaling Technology), Phospho-Akt (Thr308) (13038, Cell Signaling Technology), Pan-AKT (Ab179643, Abcam), FLAG (AP13757b, Proteintech), HRP-goat-anti-rabbit IgG (SA00001-2, Proteintech), and HRP-goat-anti-mouse IgG (SA00001-1, Proteintech).

### Immunofluorescence (IF)

Treated cells were seeded on coverslips (Sarstedt Inc. TC coverslip 13 mm ST/CS200, Fisher Scientific) were fixed with 4% PFA in PBS for 15 min at room temperature. Cells were blocked with 1% BSA dissolved in PBS for 1 h after permeabilized with 0.1% Triton X-100 in PBS for 7 min, then incubated with primary antibody anti-5-mC (28692, Cell Signaling Technology) overnight at 4 °C, followed by Fluor 488-conjugated (1:200) incubation for 1 h at room temperature. The tablets were sealed with an anti-fluorescence quencher containing DAPI (P0131, Beyotime). IF images were acquired with a laser scanning confocal microscope (Andor) equipped with Imairs Viewer software.

### Immunohistochemistry (IHC)

As previously described [60], tissue microarray and paraffin-embedded tissue samples from consenting patients were incubated overnight using the following primary antibodies: SHMT2, PTEN, and Phospho-Akt (Ser473). The IHC images were captured by a microscope (Olympus, USA). To get the relative expression (%) of the target protein, the measurement parameters collected by Image J, including stain intensity and positive staining area.

### DNA dot blot

DNA were extracted from treated cells with DNA extraction kit (DC112-01, Vazyme Biotech). The genomic DNA was diluted to 250 ng/μL in 100 μL nuclease-free water. Then, 100 μL 2× DNA denaturation buffer was added and incubated at 95 °C for 10 min following by putting 2 μL DNA with different concentration on nylon membrane. The nylon film was cross-linked with UV (1200 J/m^2^) for 15 min after drying the nylon membrane. Anti-5-mC antibody was used to incubate the membrane after blocked with 5% BSA overnight at 4 °C. Wash the membrane with Tris-buffered saline for 3 times. Images were captured by Chemiluminescence Imaging System (Tanon, Shanghai, China) after the secondary antibody incubated.

### Methylation-specific PCR (MSP)

The extracted DNA was transformed and purified using EpiArt DNA Methylation Bisulfite Kit (EM101-01, Vazyme Biotech). PCR amplified fragment with 2 × Epi Art HS Taq Master Mix (EM202-01, Vazyme Biotech) and MSP primers (Supplementary Table).

### Lung metastasis mice model

Stable BHP10-3 cells with Vector, SHMT2, shNC, shSHMT2/shNC, shNC/shPTEN, or shSHMT2/shPTEN were injected by tail vein (2×10^6^ cells per 200 μL) into BALB/c nude mice (Vital River, Beijing, China). MK-2206 (5 mg/kg) were injected through intraperitoneal every 3 days. Each group includes at least 5 mice. About 2 months later, mice lung tissues were collected after the mice were suffocated by carbon dioxide. H&E staining was used to analyze metastasis in vivo after collected lung tissue sections. The area of metastatic tumors was analyzed with Image J.

This study was reviewed and approved by the Animal Ethical and Welfare Committee. All animal studies were approved by Laboratory Animal Ethical and Welfare Committee of Shandong University Cheeloo College of Medicine, Jinan, China.

### Statistical analysis

All in vitro experiments were performed at least three times. The results were presented as the mean ± SD. Statistical analyses were performed using GraphPad Prism 7 and Microsoft Excel 2020. Student’s t-test was used to determine significant differences in the data between two experimental groups. All statistical analyses were two-tailed, *p < 0.05, **p < 0.01 and ***p < 0.001 were considered significant.

### Supplementary information

Supplement file

Original Data File

Checklist

## Data Availability

All data generated and analyzed during this study are included in this published article are available on request.
