# Supplementary material for: SHMT2 promotes papillary thyroid cancer metastasis through epigenetic activation of AKT signaling
Source: Cell Death Dis. 2024 Jan 25;15(1):87. doi: 10.1038/s41419-024-06476-1 (PMC10811326; doi:10.1038/s41419-024-06476-1)
Supplement: Supplementary file 1 — Supplement file [file 41419_2024_6476_MOESM1_ESM.docx]

**SHMT2 promotes papillary thyroid cancer metastasis through epigenetic activation of AKT signaling**

**Supplement Figures**


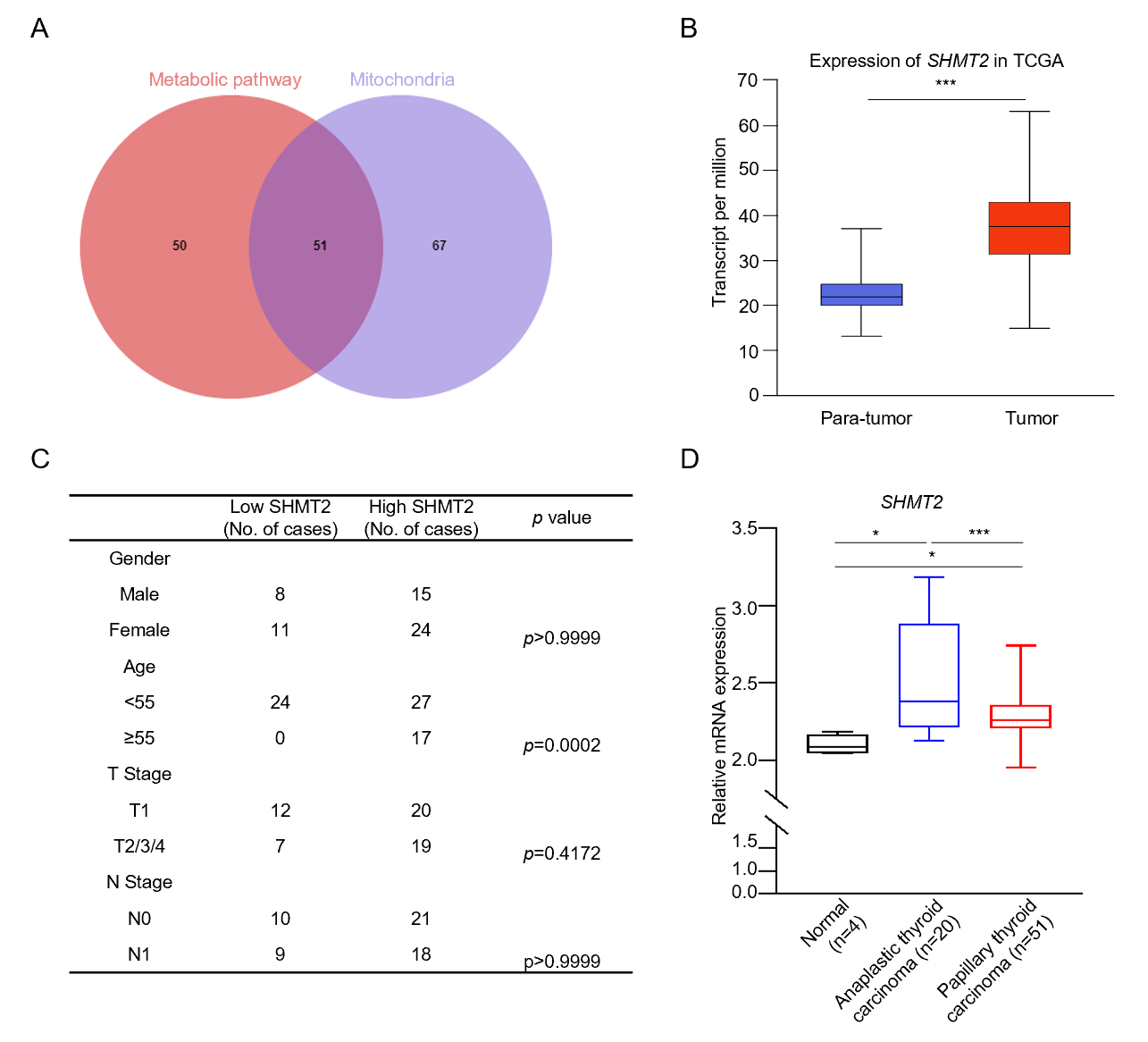


**Figure. S1 SHMT2 is highly expressed in PTC.**

A. Venn diagram showed the candidate mitochondrial proteins which highly expressed in PTC specimens.

B. Transcription levels of *SHMT2* were determined in papillary thyroid cancer and para-tumor samples from TCGA.

C. The correlation of SHMT2 expression with clinical parameters were assessed in PTC tissue microarray.

D. The mRNA levels of SHMT2 in normal, anaplastic thyroid carcinoma and papillary thyroid carcinoma clinical tissues. Data from GEO dataset (GSE27155), *: *p* < 0.05, ***: *p* < 0.001.


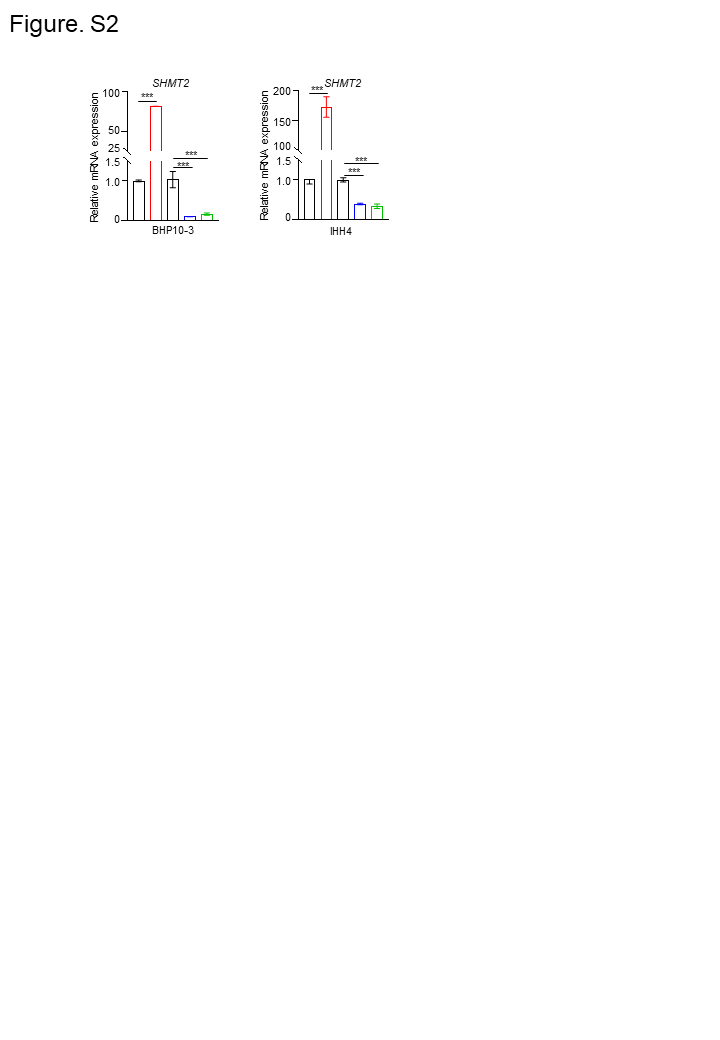


**Figure. S2. Efficiency of SHMT2 were determined in THCA cell lines.**

Relative mRNA expression levels of *SHMT2* were examined in thyroid cancer cells with SHMT2 over-expressed or knockdown by RT-qPCR. Data presented as mean±SD, n=3. ***: *p* < 0.001
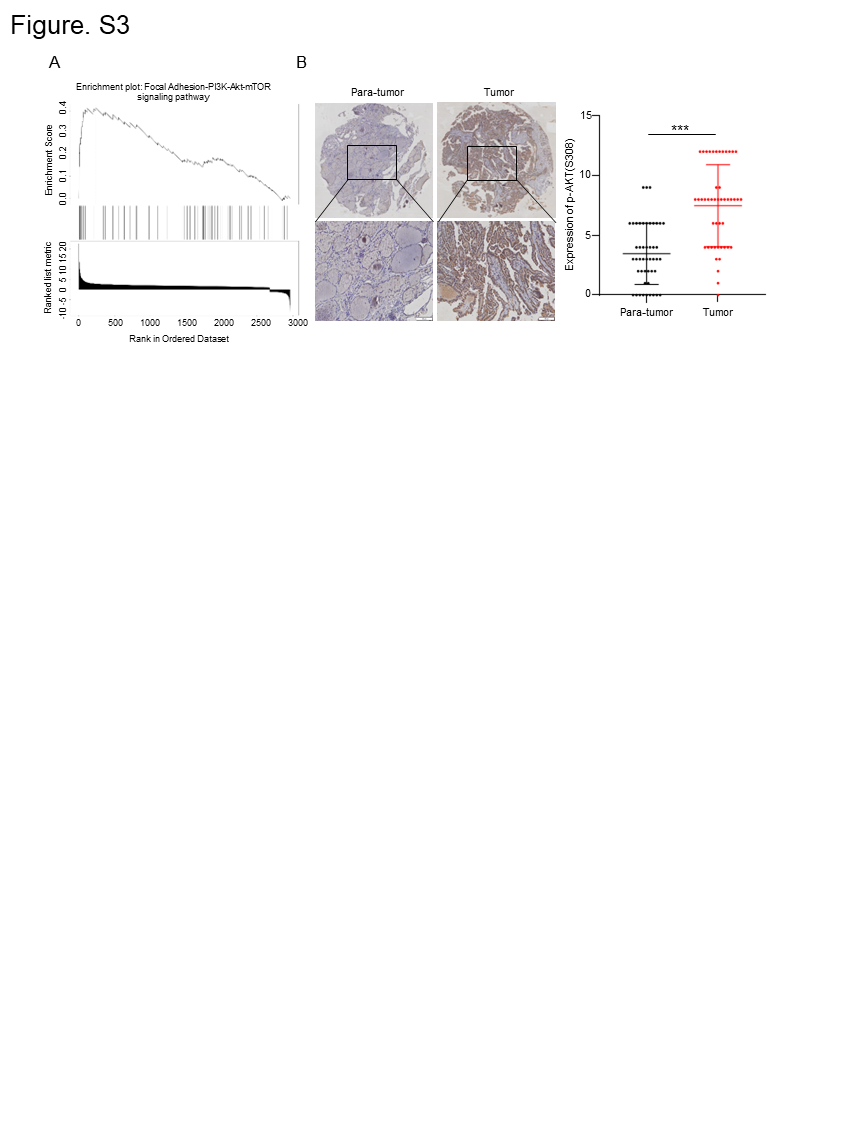


**Figure. S3 AKT signaling is activated in PTC sepecimens.**

A. GSEA was used to analyze differentially expressed proteins in PTC specimens. The enrichment of PI3K-AKT signaling was presented.

B. The expression of p-AKT (Ser 473) were detected in tumor and para-tumor tissues by IHC staining. Left: representative histological micrographs. Scale bar: 200 μm (up panel), 100 μm (bottom panel). Right: statistics of THCA microarray. Data presented as mean ± SD, n=58. ***: *p* < 0.001.


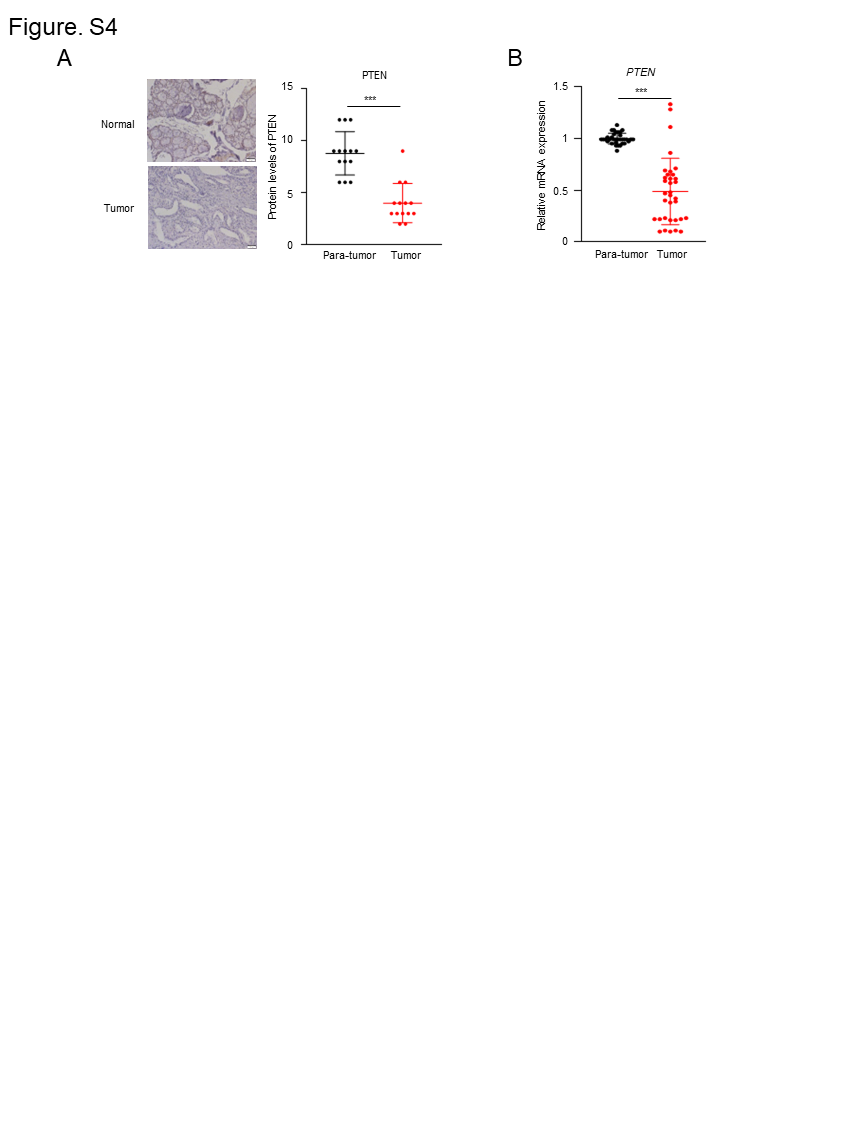


**Figure. S4 Expression of PTEN were determined in THCA specimens.**

A. The expression of PTEN were detected in tumor and para-tumor tissues by IHC staining. Left: representative histological micrographs. Scale bar: 100 μm. Right: statistics of THCA microarray. Data presented as mean ± SD, n=14. ***: *p* < 0.001.

B. The mRNA expression levels of *PTEN* were detected in para-tumor and tumor samples from Qilu Hospital by RT-qPCR. Data presented as mean ± SD, n=34. ***: *p* < 0.001.

**Supplementary table**

**Sequences of oligos were used in this work.**

| **RT-qPCR primers** | | | |
| --- | --- | --- | --- |
| **Gene** | **Forward** | **Reverse** | |
| SHMT2 | CCCTTCTGCAACCTCACGAC | TGAGCTTATAGGGCATAGACTCG | |
| β-actin | AGTTGCGTTACACCCTTTC | CCTTCACCGTTCCAGTTT | |
| E-cadherin | CGGGAATGCAGTTGAGGATC | AGGATGGTGTAAGCGATGGC | |
| N-cadherin | AGCCAACCTTAACTGAGGAGT | GAAACCGGGCTATCTGCTCG | |
| Vimentin | AGTCCACTGAGTACCGGAGAC | CATTTCACGCATCTGGCGTTC | |
| PTEN | AGGGACGAACTGGTGTAATGA | CTGGTCCTTACTTCCCCATAGAA | |
| **MSP primers** | | | |
| PTEN-Methylated-F GATGAGGTGATATACGTTGGCG | | | |
| PTEN-Methylated-R TTTACACCGCTATCGAATCACAAT | | | |
| PTEN-Unmethylated-F GGATGAGGTGATATATGTTGGTGAT | | | |
| PTEN-Unmethylated-R TTTTACACCACTATCAAATCACAATCA | | | |
| **siRNA sequences** |  | |  |
| Srcamble-sense | UUCUCCGAACGUGUCACGUTT | |  |
| Srcamble-antisense | ACGUGACACGUUCGGAGAATT | |  |
| SHMT2 1# | CUUCGAGUCUAUGCCCUAUAATT | |  |
| SHMT2 2# | CCGAAUCAACUUUGCCGUGUUTT | |  |
| PTEN 1# | CGACUUAGACUUGACCUAUAUTT | |  |
| PTEN 2# | GCCAGCYAAAGGUGAAGAUAUTT | |  |
