# Supplementary figures and images for: SHMT2 promotes papillary thyroid cancer metastasis through epigenetic activation of AKT signaling
Source: Cell Death Dis. 2024 Jan 25;15(1):87. doi: 10.1038/s41419-024-06476-1 (PMC10811326; doi:10.1038/s41419-024-06476-1)

Figure. 2B

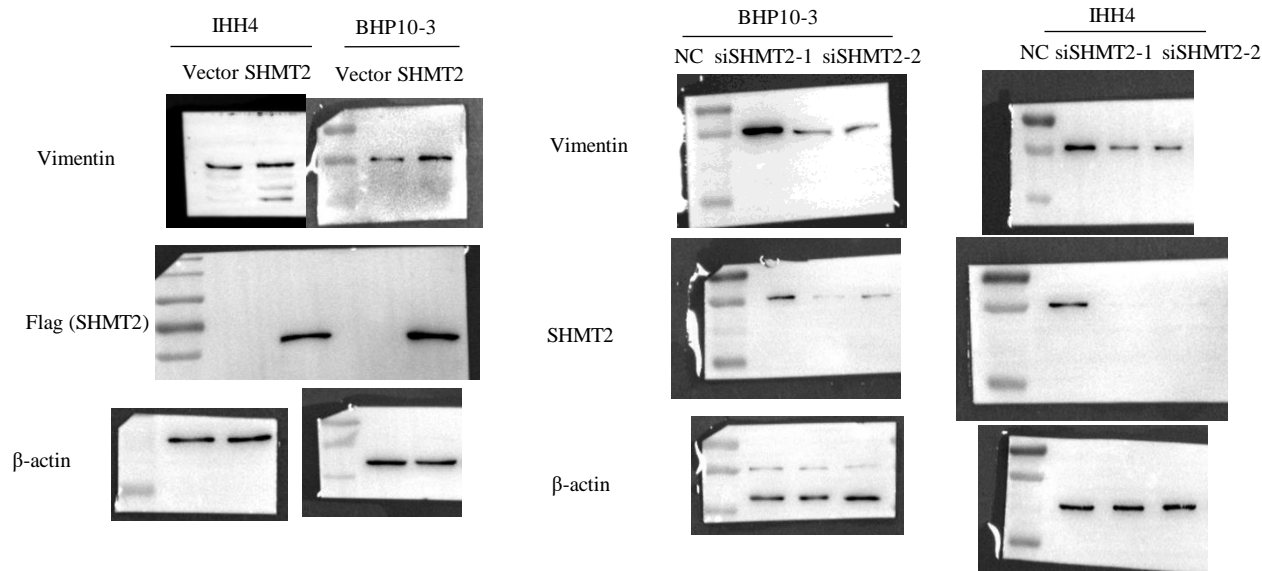

Figure. 3 C

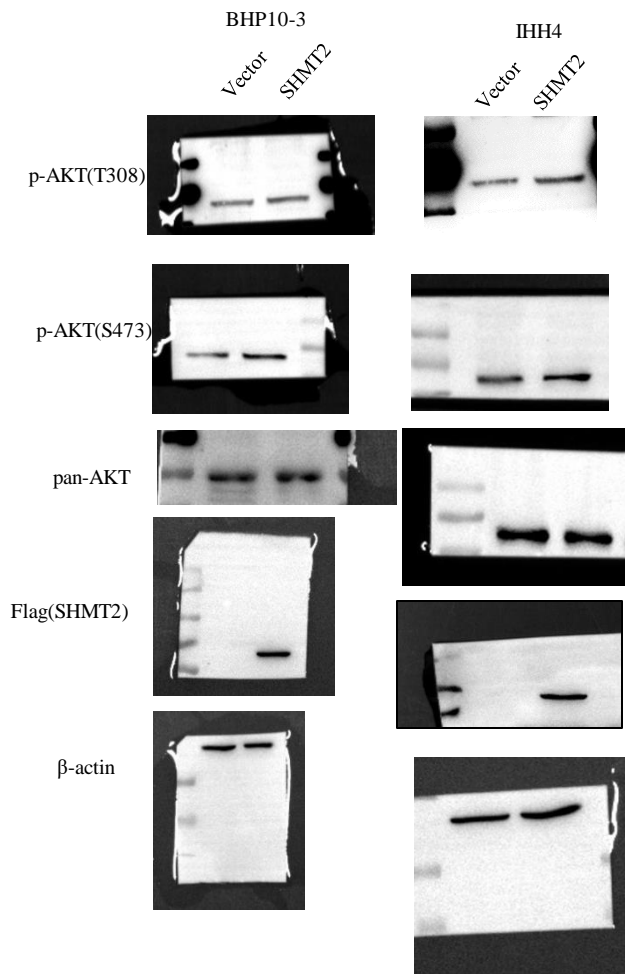

Figure. 3 D

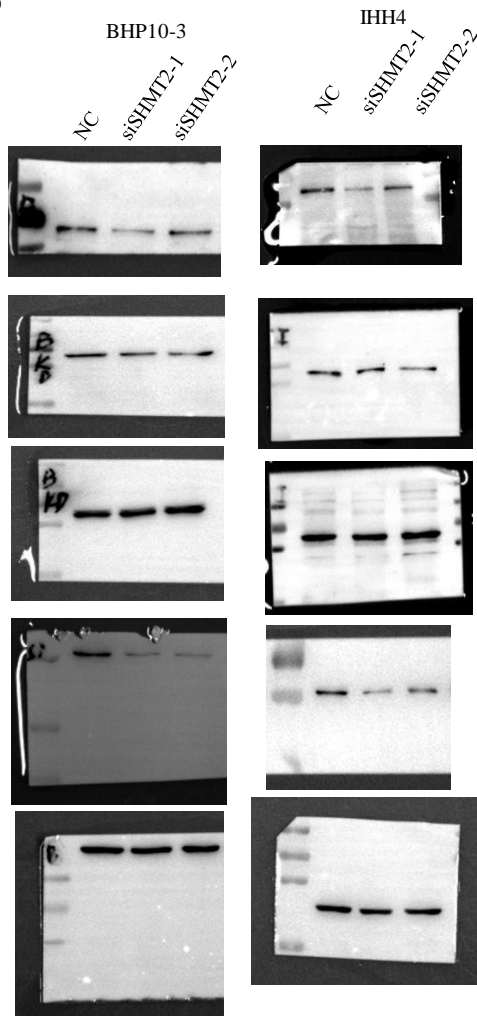

Figure. 3E

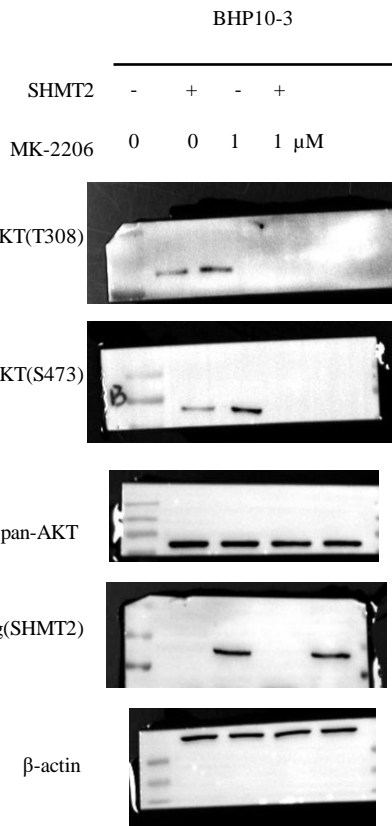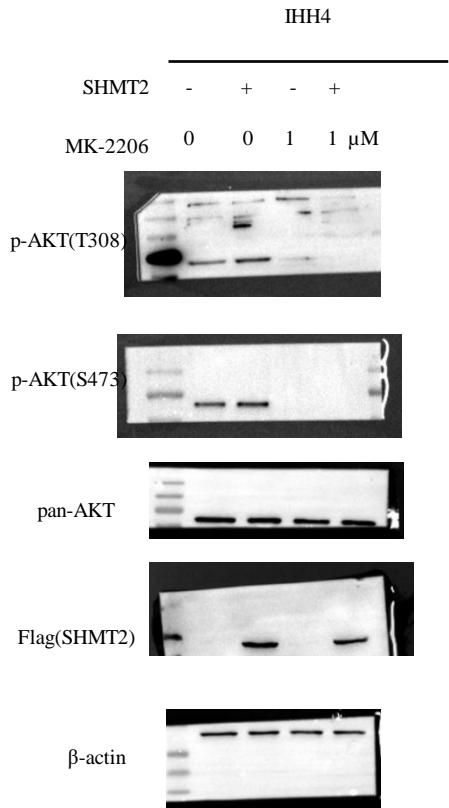

Figure. 4 A. B

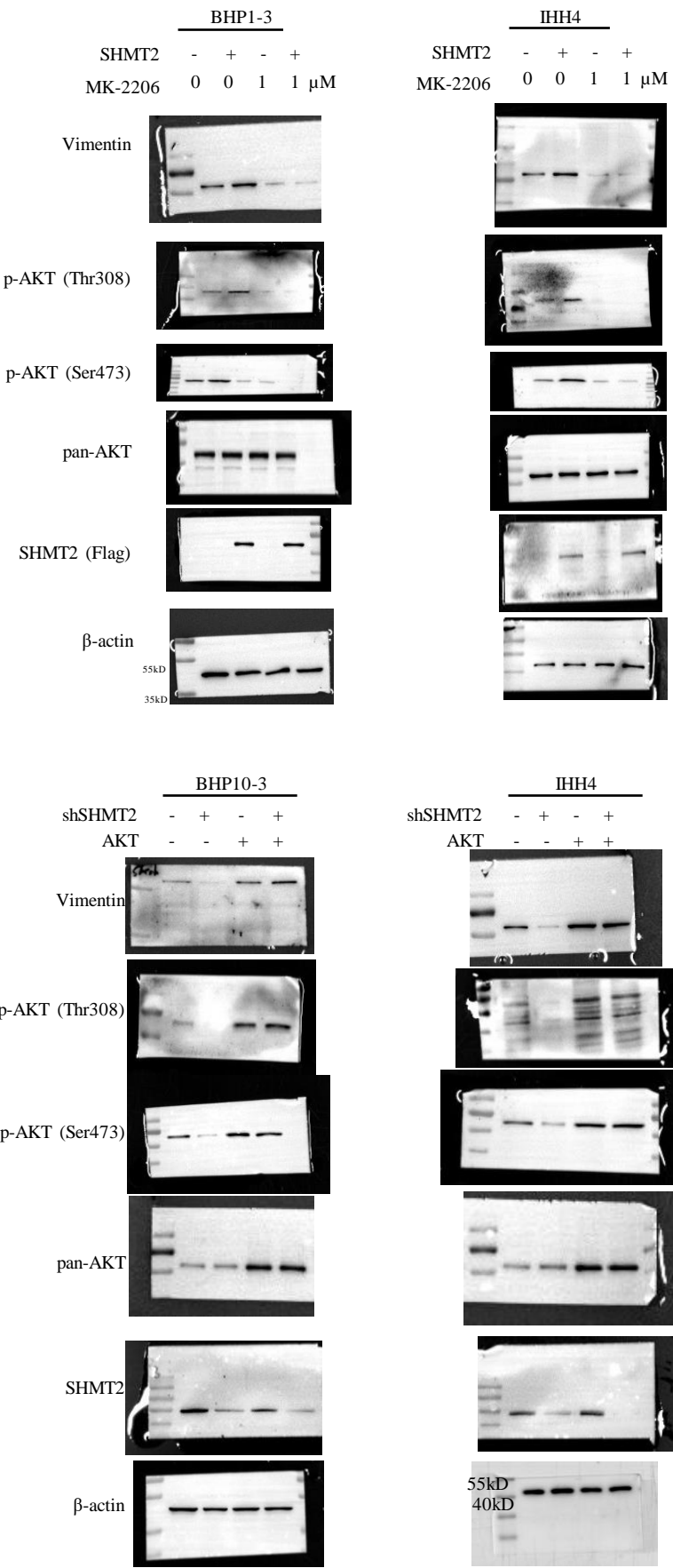

Figure. 5 D

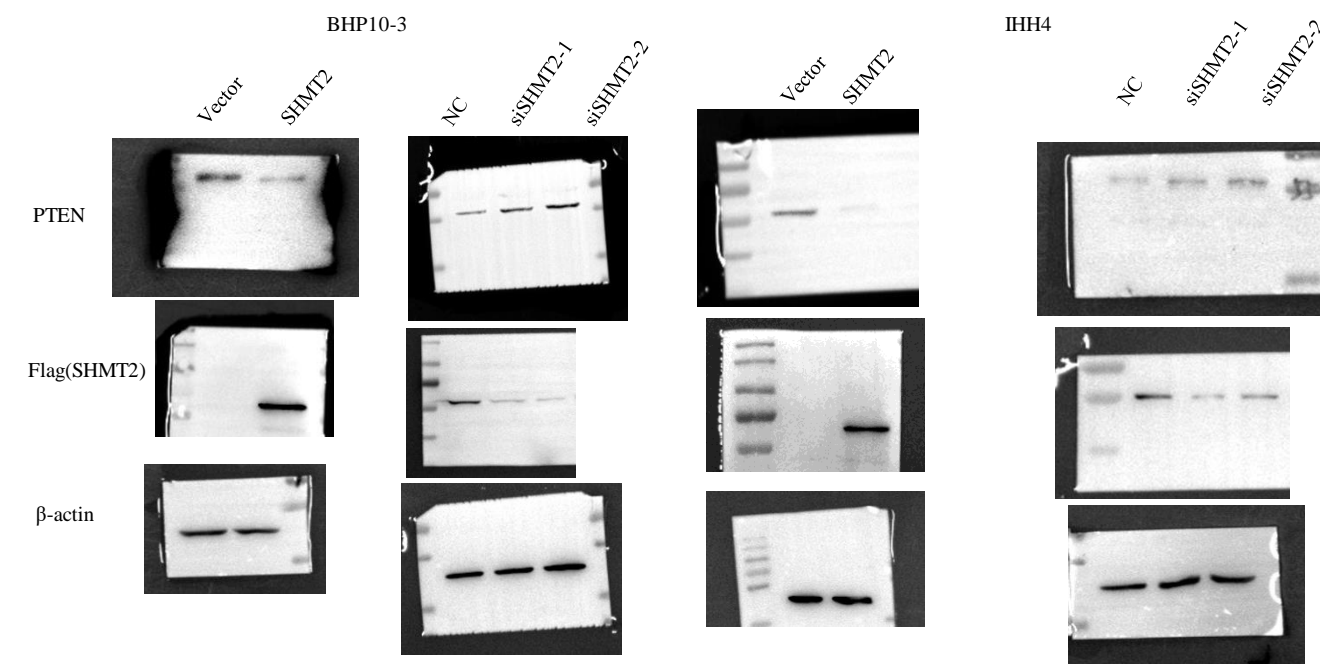

Figure. 5 E

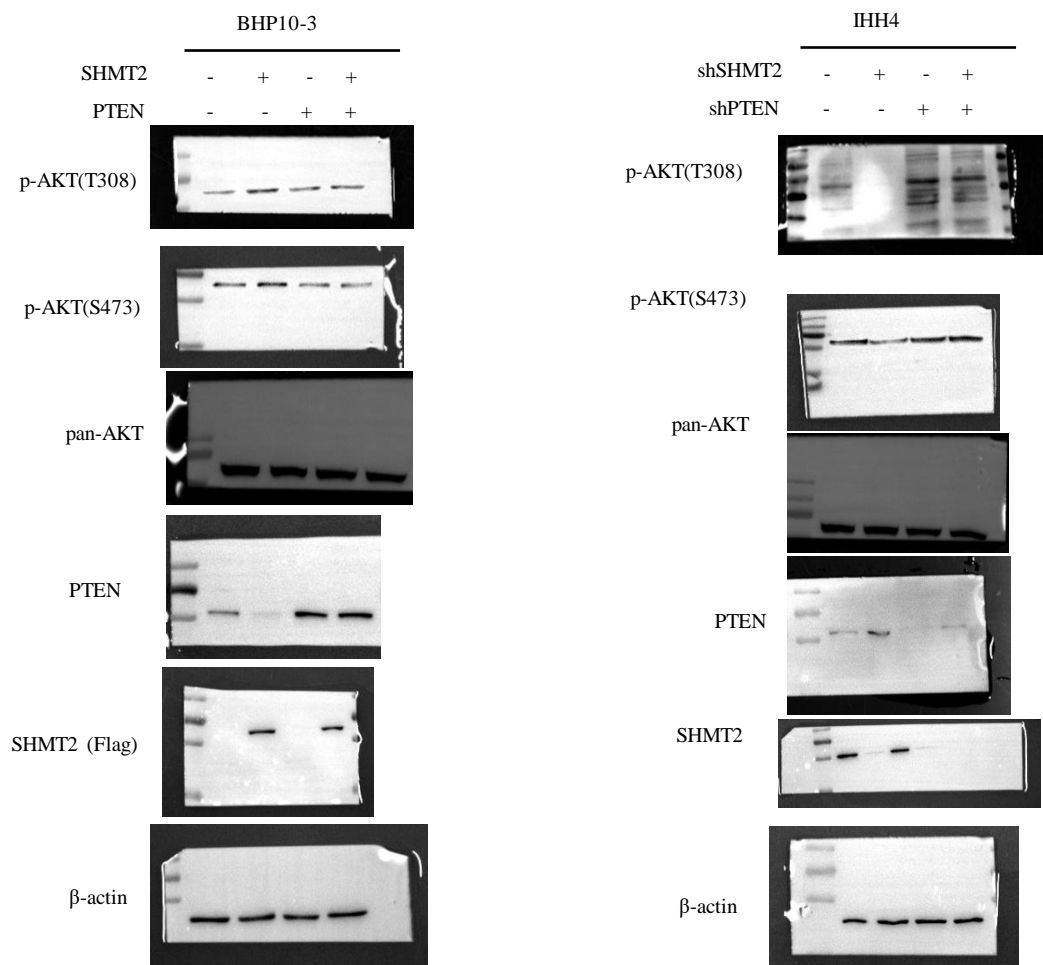

Figure. 6F

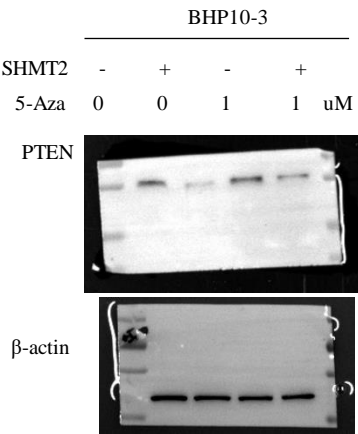

Figure. 7 A. B

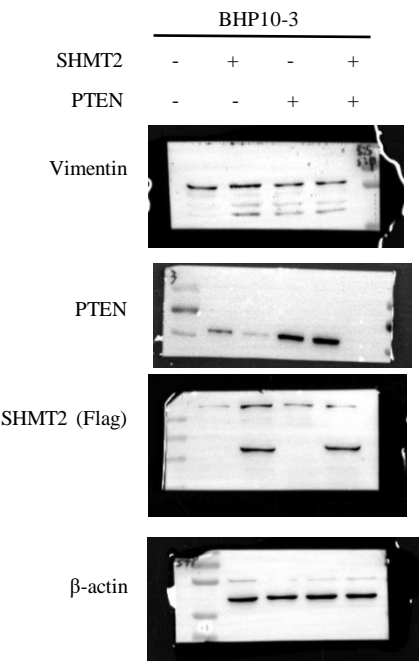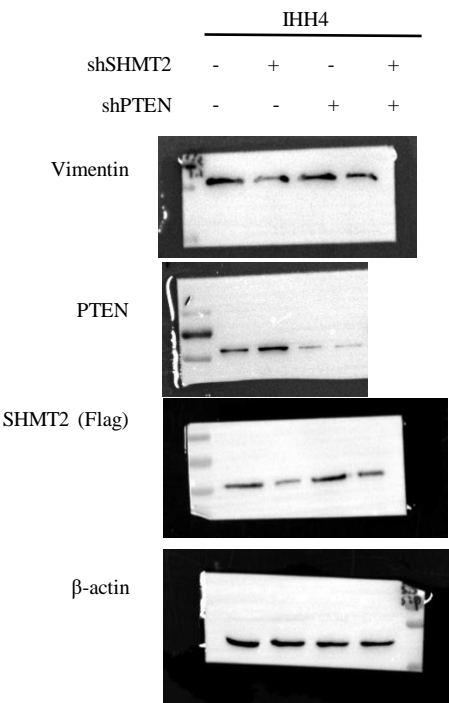

Supplement: Supplementary file 2 — Original Data File [file 41419_2024_6476_MOESM2_ESM.pdf]
